# Diagnosis and management of rectal syphilis—case report

**DOI:** 10.1093/jscr/rjac102

**Published:** 2024-11-06

**Authors:** Zeeshan Afzal, Adam Hussein, M O’Donovan, David Bowden, Richard J Davies, Simon Buczacki

**Affiliations:** Cambridge Colorectal Unit, Cambridge University Hospital NHS Foundation Trust, Cambridge, UK; Cambridge Colorectal Unit, Cambridge University Hospital NHS Foundation Trust, Cambridge, UK; Department of Histopathology & Cytology, Cambridge University Hospital NHS Foundation Trust, Cambridge, UK; Department of Radiology, Cambridge University Hospital NHS Foundation Trust, Cambridge, UK; Cambridge Colorectal Unit, Cambridge University Hospital NHS Foundation Trust, Cambridge, UK; Cambridge Colorectal Unit, Cambridge University Hospital NHS Foundation Trust, Cambridge, UK; Nuffield Department of Surgical Sciences, University of Oxford, Headington, Oxford, OX3 7DQ, UK

**Keywords:** case report, rectal, syphilis, *Treponema pallidum*, chancre

## Abstract

The incidence and prevalence of syphilis are rising worldwide. Rectal syphilis is a rare condition with few reported cases in the literature and therefore often missed from differential diagnosis of atypical anorectal ulceration. We report a case of a 64-year-old male who presented with change in the bowel habit and a palpable rectal mass on examination. Colonoscopy revealed a small, ulcerated lesion in the rectum. However, histopathological analysis and radiological assessments were inconclusive. A cutaneous ulceration prompted a repeat biopsy and staining for spirochaetes, which was diagnostic of syphilitic proctitis. He was successfully treated with first line antibiotics via the Genitourinary Medicine clinic. With its increasing incidence, syphilis should be considered as a potential diagnosis of atypical anorectal ulceration. A complete sexual history including relevant risk factors should be taken and a full clinical examination performed actively looking for signs and symptoms of disease.

## Introduction

Syphilis is a well-known sexually transmitted infection (STI) that can cause serious health problems if left untreated. The incidence and prevalence of syphilis are rising worldwide; however, its manifestation in rectum, also known as rectal syphilis, is a rare condition. It was first reported in the early 1940s and since then, only 50 cases have been reported in the literature so far to the best of our knowledge, hence it is often missed, leading to delayed treatment. This case report highlights the significance of considering rectal syphilis in the differential diagnosis of atypical anorectal ulceration and how this can mimic rectal cancer.

## Case presentation

A 64-years-old male was reviewed in colorectal cancer clinic for significant change in the bowel habit and a palpable rectal mass on examination. Apart from diverticular disease, he was fit and well and not on any regular medications. There was no family history of colorectal cancer of note. Patients did have sexual history of multiple same gender (male) partners; however, this was only picked up later in the due course.

Computed tomography (CT) scan of pelvis showed mild thickening of the rectum with local lymphadenopathy; however, magnetic resonance imaging (MRI) of the pelvis was normal.

**Figure 1:**
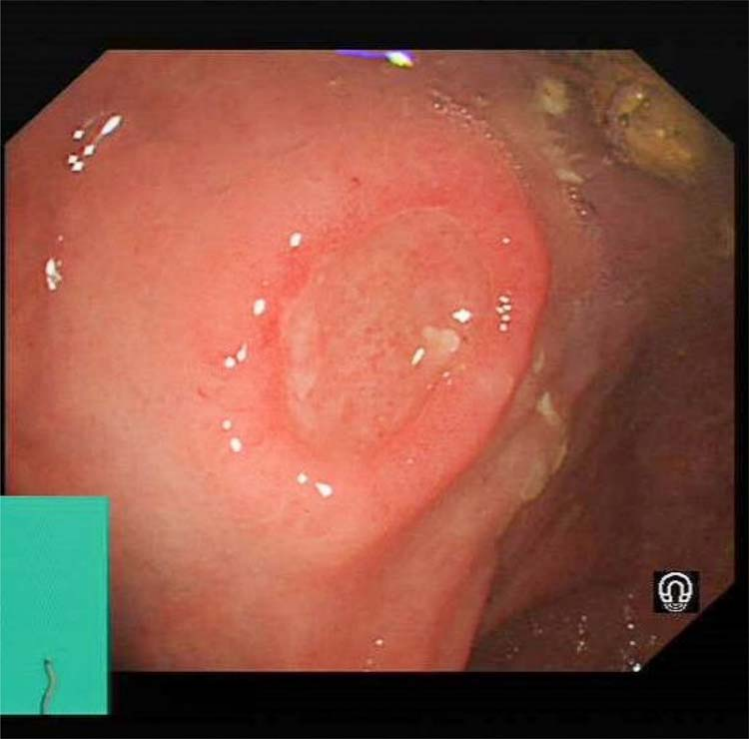
Colonoscopy images of a 64-year-old male with rectal mass. The images show a 10 mm elevated lesion with central ulceration in the rectum, ~5–6 cm from the anal verge.

Colonoscopy revealed a 10 mm elevated lesion with central ulceration in the rectum, ~5–6 cm from the anal verge. The appearances were not typical of an adenocarcinoma, and biopsies were taken which showed non-specific active chronic inflammation (Fig. 1).

The patient was discussed in the lower gastrointestinal multi-disciplinary team meetings, and a repeat flexible sigmoidoscopy was recommended.

Subsequently, patient developed new non-specific lesions on his arms, penis and thighs, and was reviewed in a local Genitourinary Medicine clinic. Blood tests showed positive syphilis serology: enzyme immunoassays (EIA) positive, pallidum particle agglutination assay positive. Rapid plasma reagin (RPR) was positive at a titer of 1:128. Although tests for gonorrhea, chlamydia, human immune deficiency virus and hepatitis B and C virus were negative.

**Figure 2:**
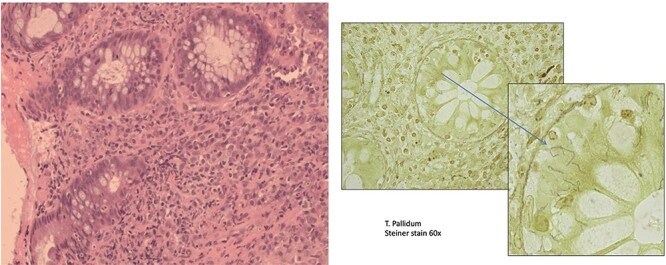
Rectal syphilis in a 64-years-old male with rectal mass (A). This section shows active chronic proctitis (Hematoxylin & Eosin stain, ×20 magnification) H + E 20×. (B) *Treponema pallidum* (Steiner stain, 60× magnification).

Repeat rectal biopsies showed moderately active chronic proctitis. Given the intervening history, a Steiner stain was performed, which showed spirochetes (*Treponema pallidum*), diagnostic of syphilitic proctitis (Fig. 2).

He was treated with 2 weeks course of oral Penicillin G Benzathine antibiotic. An endoscopic follow-up 3 months after the treatment, confirmed the resolution of rectal ulcer.

## Discussion

**Figure 3:**
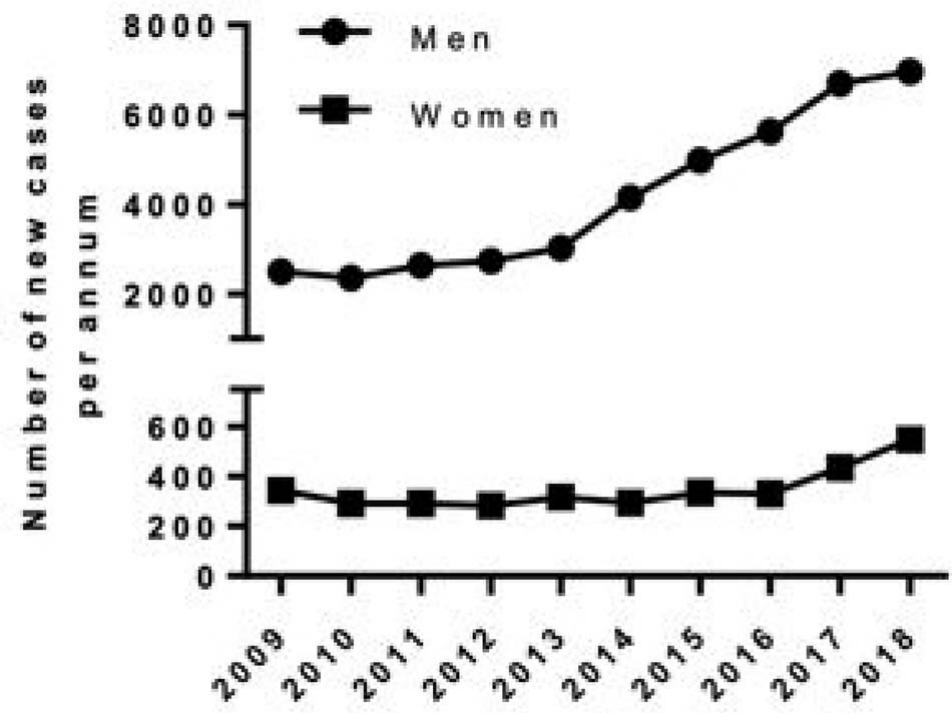
A graphical representation of number of new cases of syphilis by gender in England from 2009 to 2018.

There is a steady increase in prevalence of syphilis in the UK and worldwide (Fig. 3). This follows a decline in its prevalence between the 1980s and early 1990s, which has been attributed to a behavioral change in relation to the HIV pandemic. In the UK, from 1997 to 2007 a 12-fold increase from 301 cases in 1997 to 3762 cases in 2007 was observed [1, 2]. The prevalence of syphilis in London rose by 22% between 2014 and 2015 [3].

A total of 5042 cases of syphilis were reported in England in 2015, 90% being in men; with men who have sex with men (MSM) being a particularly high-risk group [3]. There were 7541 diagnoses of syphilis reported in 2018, a 5% increase since 2017 [4]. A similar trend was observed in USA, where a total of 30 644 cases of syphilis were reported in 2017 representing a 10.5% increase compared with 2016 [5].

Syphilis is a well-known STI that can lead to serious health issues if left untreated. The causative agent is a spirochete bacterium, *T. pallidum*. Its mode of transmission is direct contact with an infectious lesion. It can take up to 21 days (range 10–90 days) for first symptoms to appear. If untreated it may progress from primary and secondary stage to tertiary and latent stages [6, 7].

Chancre is the most characteristic feature of primary syphilis; described as a painless ulcer with a clean base and sharply marginated border, which can be associated with localized lymphadenopathy [8]. These typically heal within 3–6 weeks. Diagnosis can be very difficult if these lesions occur at occult areas such as the rectum.

Secondary syphilis is characterized by multisystem involvement, including rash, fever, lymphadenopathy and patchy lesions on the oral mucosa. Tertiary syphilis can be fatal but it is rare. It can present 10–30 years after the initial infection and symptoms vary depending on the system affected. There is a latent stage of syphilis whereby, without treatment, the patient will continue to be infected despite no visible signs or symptoms of the disease [6, 7].

Management should be via a specialist genito-urinary medicine (GUM) clinic. Patients should be provided with information on the condition. Syphilis testing includes a blood test (syphilis serology) and a virology swab from any active lesions. Screening for other STIs is recommended. Syphilis serology includes treponemal EIA, *T.* pallidum hemagglutination test (TPHA), *T. pallidum* particle agglutination test (TPPA), fluorescent treponemal antibody absorbed test (FTA-abs) and the *T. pallidum* recombinant antigen line immunoassay [9, 11]. If all test results are negative, repeat serology testing at 6 and 12 weeks is advised. If any of the test is positive, the patient should be treated with antibiotics.

Current guidelines from British Association for Sexual Health and HIV suggest benzathine penicillin G 2.4 million Units intramuscular single dose for early syphilis [9, 10].

## Conclusion

With the current increasing rates, syphilis should be considered as a potential diagnosis of atypical anorectal ulceration. Rectal syphilis can mimic rectal cancer as in our case and since it is rare, it is often missed, leading to delayed treatment. A complete sexual history including the risk factors should be taken and a full systematic head to toe examination carried out actively looking for signs of disease. Targeted investigation such as syphilis serology and endoscopic biopsy of the anorectal lesion can lead to earlier diagnosis, allowing a tailored management plan including early involvement of the infectious diseases and GUM team. This can save time and health resources while ensuring the correct treatment is started in a timely fashion and providing reassurance to the patient.

## Data Availability

Not applicable. All authors are happy for data to be shared and reused.
